# Weight changes from early to middle adulthood and cardiometabolic multimorbidity later in life among middle-aged and older adults: a retrospective cohort study from the NHANES 1999-2018

**DOI:** 10.3389/fendo.2024.1306551

**Published:** 2024-02-19

**Authors:** Fayun Zhao, Qiang Zhao, Huandong Wang, Kun Wang, Shenke Kong, Peiyao Ma, Xin Wang

**Affiliations:** Department of Cardiology, Henan Provincial Chest Hospital, Zhengzhou, China

**Keywords:** body mass index, body weight changes, obesity, cardiometabolic diseases, cardiometabolic multimorbidity

## Abstract

**Background:**

Weight gain in adulthood can influence the development of diabetes and cardiovascular diseases. It is speculated that weight gain is related to cardiometabolic multimorbility (CMM). This study was designed to examine the relationships between weight changes from early to middle adulthood and the risk of CMM.

**Methods:**

Data of the National Health and Nutrition Examination Survey (NHANES) 1999-2018 cycles were analyzed in the present study. Weights at age 25 years and 10 years before recruitment were self-reported and were used to define five weight change patterns including stable normal, maximum overweight, obesity to non-obesity, non-obesity to obesity, and stable obesity patterns. Meanwhile, absolute weight changes were classified into five groups: weight loss≥ 2.5 kg, weight change within 2.5 kg, 2.5 kg≤ weight gain < 10.0 kg, 10.0 kg≤ weight gain < 20.0 kg, and weight gain≥ 20.0 kg. CMM was defined as the coexistence of two or three of diabetes, coronary heart disease (CHD), and stroke.

**Results:**

A total of 25,994 participants were included. Across adulthood, compared to stable normal weight, maximal overweight, obesity to non-obesity, non-obesity to obesity, and stable obesity were consistently associated with increased risks of diabetes, CHD, and CMM. For instance, stable obesity was respectively related to 358.0% (*HR*: 4.58, *95% CI*: 4.57, 4.58), 88.0% (*HR*: 1.88, *95% CI*: 1.88, 1.88), and 292.0% (*HR*: 3.92, *95% CI*: 3.91, 3.92) higher risks of diabetes, CHD, and CMM. Meanwhile, any account of weight loss and gain was linked to higher risks of diabetes, CHD, and CMM than weight change within 2.5 kg. However, participants with maximum overweight had a decreased incidence of stroke (*HR*: 0.85, *95% CI*: 0.85, 0.86), and weight loss ≥ 2.5 kg and weight gain ≥ 2.5 and <20 kg were also related to a lower risk of stroke. J-shaped or U-shaped associations of absolute weight changes with the risks of diabetes, CHD, and CMM were observed.

**Conclusions:**

Maintaining a stable normal weight can benefit more from the prevention of diabetes, CHD, and CMM. Both weight gain and loss across adulthood were accompanied by increased risks of diabetes, CHD, and CMM.

## Introduction

Obesity has been an emerging epidemic in the past five decades. The prevalence of obesity reached 11% and 15% for men and women respectively in 2014, rising from 5% and 8% respectively in 1980 (1). In the US, the prevalence of obesity was 42.4% in 2018 (2, 3). Obesity is associated with diabetes, cardiovascular diseases (CVD), and mortality. It is estimated that about 4 million deaths were attributed to obesity in 2015 (4). Therefore, the burden of diseases attributed to obesity-related diseases will be a major challenge.

On the other hand, cardiometabolic diseases (CMDs)—defined as diabetes, coronary heart disease (CHD), and stroke—have also become a global health problem. There were more than 463 million people suffering from diabetes, 197 million people suffering from CHD, and 12.2 million people suffering from stroke worldwide (5, 6). As the population ages, cardiometabolic multimorbidity (CMM), defined as the coexistence of at least two CMDs, will be a common phenomenon (7). A previous study showed that an increase per 5 kg/m^2^ in BMI was related to a 1.9 higher risk of CMM (7). However, this study only involved a single measurement of BMI not the dynamic changes in body weight over time. It is documented that weight gain is common from early to middle adulthood and is linked to increased risks of diabetes, CVD, hypertension, and mortality (1, 8–11). Therefore, it is speculated that weight gain is related to CMM. However, few studies were conducted to evaluate the relationship between weight gain across adulthood with the risk of CMM. Therefore, the purpose of this study is to dissect the relationship between weight changes from early (age 25 years) to middle adulthood (mean age 47 years) and the risk of new-onset CMDs and CMM using the data of National Health and Nutrition Examination Survey (NHANES).

## Materials and methods

### Study population

Data analyzed in the present study were derived from the NHANES 1999-2018 cycles. NHANES is a series of ongoing cross-sectional surveys and is designed to assess the health and nutritional status of the non-institutionalized population in the US. Data from questionnaires, physical examinations, and laboratory tests were collected by trained interviews. A multistage stratified cluster random sampling method was used in continuous NHANES surveys, which started in 1999, and thereafter in 2-year cycles. Details of NHANES are found at the NHANES website (https://www.cdc.gov/nchs/nhanes/index.htm) (12).

**Figure 1:**
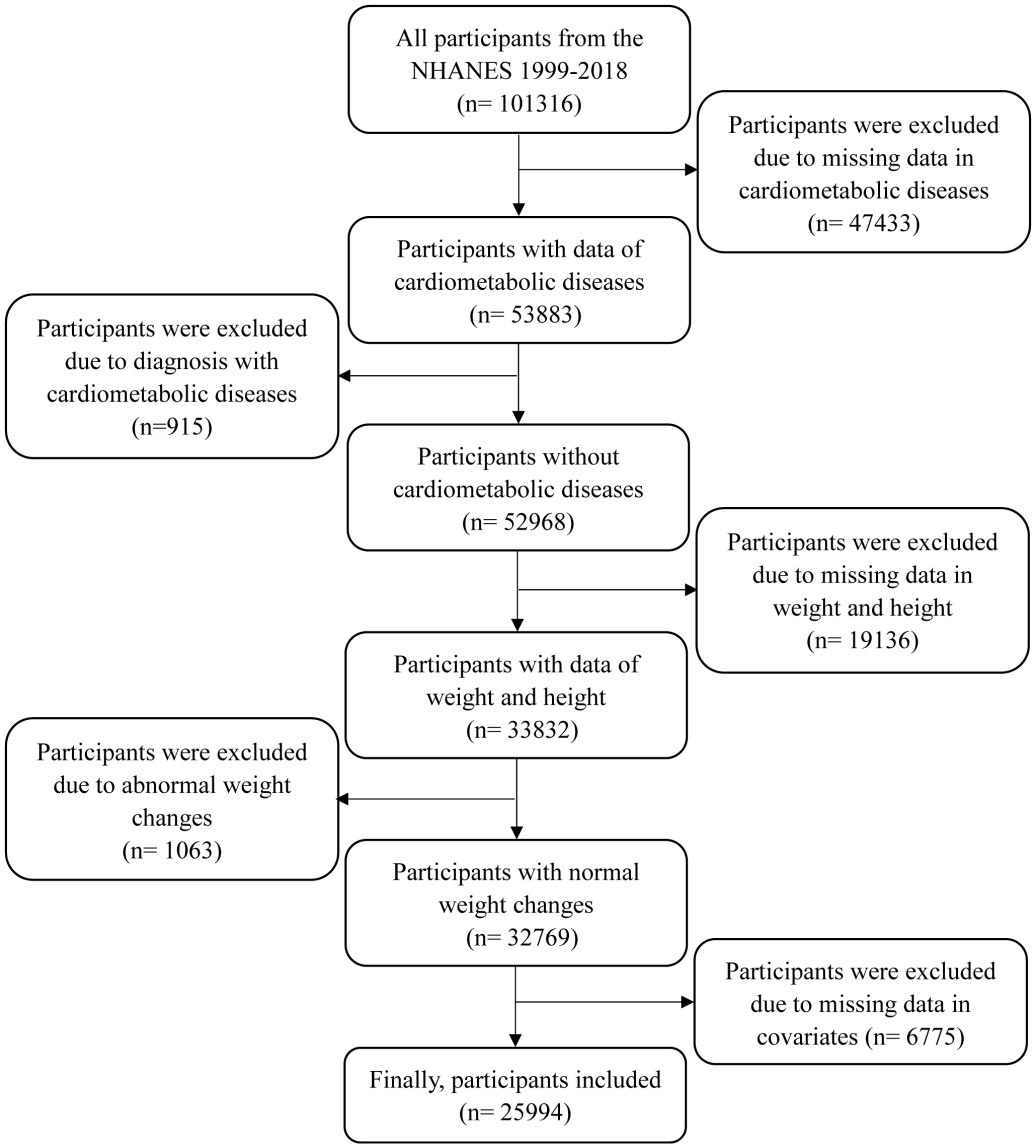
The process of participant selection.

Since eligible participants for weight at 10 years before recruitment were aged≥ 36 years, this study incorporated data on participants aged≥ 36 years at the time of the survey. There were 47,433 participants excluded due to missing data in diabetes, CHD, and stroke, 915 participants due to diagnosis with CMD at baseline, 19,136 participants due to missing data in weight and height, 1063 participants due to abnormal weight changes, and 6775 participants due to missing data in covariates (as shown in **Figure 1**). Finally, a total of 25,994 participants were included in this study. This study was approved by the NHANES Institutional Review Board (protocols Numbers: NHANES Protocol #2011-17 and NHANES Protocol #2018-01). Written informed consent was obtained from all participants.

### Assessments of weight changes

In the NHANES, participants were required to recall weight and height at age 25 years as well as weight 10 years before the survey. BMI at age 25 years and BMI at 10 years before recruitment (mean age: 47 years, range: 26-75 years, interquartile range: 36-58 years) were calculated as weight (kg) divided by the square of height (m^2^). For participants aged< 50 years at the time of the survey, weight at age 25 years and height at the examination were used to calculate BMI at age 25 years. Otherwise, weight and height at age 25 years were used to calculate BMI at age 25 years. BMI at 10 years before recruitment was calculated using weight and height at 10 years before recruitment (10). According to a previous study, BMI at the two time points were categorized into underweight or normal weight, overweight, and obesity using cut-off values of 25.0 kg/m^2^ and 30.0 kg/m^2^ (9). According to BMI at age 25 years and 10 years before recruitment, five weight-change patterns were identified as follows: stable normal pattern for underweight or normal weight at both times, maximum overweight pattern for overweight at either time, obesity to non-obesity pattern for obesity at age 25 years and non-obesity at 10 years before recruitment, non-obesity to obesity pattern for non-obesity at age 25 years and obesity at 10 years before recruitment, and stable obesity for obesity at both times (13). Meanwhile, absolute weight change between age 25 years and 10 years before recruitment was classified into five groups: weight loss≥ 2.5 kg, weight change within 2.5 kg (reference group), 2.5 kg≤ weight gain < 10.0 kg, 10.0 kg≤ weight gain < 20.0 kg, and weight gain≥ 20.0 kg (14, 15).

### Outcomes

**Figure 2:**
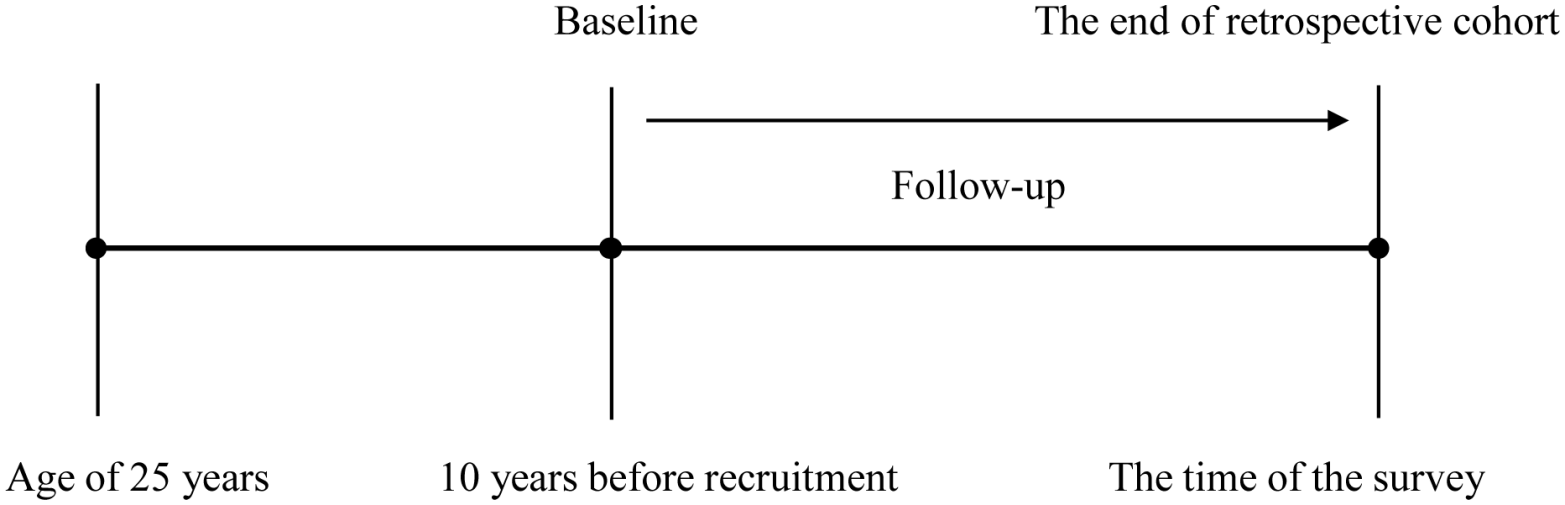
The schematic diagram of retrospective cohort study.

According to previous studies, this study was designed as a retrospective cohort from the cross-sectional data, as shown in **Figure 2** (9, 10, 16). Baseline was defined as 10 years before recruitment. Incident CMDs were identified if participants reported that they had been diagnosed with one of the CMDs by a physician. CMM was defined as two or three of CMDs. When single CMDs as the end-event, participants with the corresponding CMDs at baseline were excluded. The time of follow-up was defined as the interval between baseline and the age, at which single CMDs were established. When CMM was the end-event, participants with two or three of CMDs at baseline were excluded. The time of follow-up was defined as the interval between baseline and the age, at which the second CMD was established. Therefore, datasets for single CMDs and CMM may be different in sample size.

### Statistical analysis

Characteristics of all participants were expressed as mean ± standard deviation (SD) for continuous variables with a normal distribution, median (range interquartile) for abnormal distribution, and frequency (percent) for categorized variables. *Analysis of variance*, *Kruskal-Wallis* test, and *chi-square* test were respectively employed to compare the differences across weight change patterns and absolute weight changes for continuous variables with normal distribution, for ones with abnormal distribution, and for categorized variables. The hazard ratios (*HRs*) and 95% confidence intervals (95% *CIs*) were used to evaluate the associations of weight changes with the risks of single CMDs and CMM using Cox proportional hazards models. The proportional hazards assumption was examined by including a cross-product of follow-up time and weight change patterns in Cox proportional hazards models. If likelihood ratio tests comparing models with and without this variable were not significant, the proportional hazards assumption held. The associations of weight change patterns and absolute weight changes with CMM were further stratified by age (< 60 years and ≥ 60 years) and sex (males and females). Covariates including age, sex, race, education level, current smoking, current alcohol drinking, family income-poverty ratio level, marital status, family history of diabetes or CVD, MET, healthy eating index scores, and history of hypertension were adjusted with CMM as the end-event. When one of CMDs as the end-event, the other two CMDs were additionally adjusted. When absolute weight changes were analyzed, height at examination was additionally adjusted. Furthermore, the sampling weight was used to adjust for non-response bias and over-sampling. All the variables with missing data were imputed using the Multivariate Imputation by Chained Equations (*mice*) package in the statistical program R (version 3.5.1) to explore the robustness of the main results. The non-linear associations of absolute weight changes with the risks of single CMDs and CMM were further examined using a restricted cubic spline with four knots. All analyses were performed using SAS 9.4 (SAS Institute Inc., Cary, NC, USA.). A two-tailed *P*≤ 0.05 indicated a statistical significance.

## Results

### Characteristics of participants across weight change patterns

There were 25,994 participants (mean of age: 47.26 ± 13.55 years) in this study, including 13,073 men and 12,921women. The averages of BMI at age 25 years and 10 years before recruitment were 23.53 ± 4.41 kg/m^2^ and 27.39 ± 5.71 kg/m^2^, respectively. The mean of absolute weight change was 9.34 ± 11.38 kg. The proportions of the five weight change patterns were respectively 36.00% for stable normal, 37.37% for maximum overweight, 0.97% for obesity to non-obesity, 19.31% for non-obesity to obesity, and 6.34% for stable obesity. Meanwhile, the distributions of absolute weight change were 26.53% for weight loss ≥ 2.5 kg, 5.73% for weight change within 2.5 kg, 30.34% for weight gain ≥ 2.5 and <10 kg, 21.90% for weight gain ≥ 10 and < 20 kg, and 15.50% for weight gain ≥ 20 kg. During 10 years of follow-up, the cumulative incidences of CMDs were 14.24% for diabetes, 6.42% for CHD, and 3.08% for stroke, and CMM was 4.81%.

The distributions of characteristics across weight change patterns are shown in **Table 1**. Significant differences were observed in all characteristics across weight change patterns. Meanwhile, **Supplementary Table 1** shows the characteristics of participants across absolute weight change, and significant differences were observed in all characteristics.

**Table 1:** Characteristics of participants in the NHANES 1999–2018 according to weight change patterns from age 25 years to 10 years before recruitment.

Characteristics	Total sample (n= 25994)	Stable normal (n= 9359)	Maximum overweight (n= 9714)	Obesity to non-obesity (n= 252)	Non-obesity to obesity (n= 5020)	Stable obesity (n=1649)	*P*
Age (years) ^※^	47.26 ± 13.55	44.64 ± 13.50	48.81 ± 13.54	45.45 ± 13.15	51.54 ± 12.29	40.24 ± 11.49	<0.001
MET (MET-min/week)^§^	588 (14, 2200)	661 (35, 2240)	600 (21, 2300)	530 (5, 2400)	1991 (405,6089)	540 (0, 2560)	<0.001
HEI score^※^	53.24 ± 13.77	53.64 ± 14.24	53.55 ± 13.57	52.07 ± 14.71	52.90 ± 13.26	50.36 ± 13.26	<0.001
BMI at age 25 years (kg/m^2^) ^※^	23.53 ± 4.41	20.77 ± 2.04	23.50 ± 2.78	32.56 ± 3.77	24.62 ± 3.12	34.66 ± 4.80	<0.001
BMI at 10 years before recruitment (kg/m^2^) ^※^	27.39 ± 5.71	22.29 ± 1.94	27.14 ± 1.56	27.18 ± 2.16	33.82 ± 3.70	38.30 ± 6.59	<0.001
Absolute weight change (kg) ^※^	9.34 ± 11.38	3.36 ± 5.21	8.64 ± 8.15	-12.46 ± 8.85	22.97 ± 12.11	9.21 ± 13.98	<0.001
Sex^#^							<0.001
Male	13073 (50.29)	3772 (40.30)	5747 (59.16)	145 (57.54)	2552 (50.84)	857 (51.97)	
Female	12921 (49.71)	5587 (59.70)	3967 (40.84)	107 (42.46)	2468 (49.16)	792 (48.03)	
Race^#^							<0.001
Mexican-American	3862 (14.86)	1089 (11.64)	1615 (16.63)	62 (24.60)	834 (16.61)	262 (15.89)	
Other Hispanic	1885 (7.25)	643 (6.87)	792 (8.15)	17 (6.75)	339 (6.75)	94 (5.70)	
Non- Hispanic White	13097 (50.38)	4896 (52.31)	4843 (49.86)	110 (43.65)	2528 (50.36)	720 (43.66)	
Non- Hispanic Black	5276 (20.30)	1739 (18.58)	1859 (19.14)	56 (22.22)	1129 (22.49)	493 (29.90)	
Other Race	1874 (7.21)	992 (10.60)	605 (6.23)	7 (2.78)	190 (3.78)	80 (4.85)	
Education level^#^							<0.001
Less than high school	6382 (24.55)	2094 (22.37)	2521 (25.95)	94 (37.30)	1306 (26.02)	367 (22.26)	
High school or equivalent	6182 (23.78)	2131 (22.77)	2290 (23.57)	54 (21.43)	1271 (25.32)	436 (26.44)	
College or above	13430 (51.67)	5134 (54.86)	4903 (50.47)	104 (41.27)	2443 (48.67)	846 (51.30)	
Current smoking^#^							<0.001
No	12959 (49.85)	4628 (49.45)	4788 (49.29)	94 (37.30)	2562 (51.04)	887 (53.79)	
Yes	13035 (50.15)	4731 (50.55)	4926 (50.71)	158 (62.70)	2458 (48.96)	762 (46.21)	
Current alcohol drinking^#^							<0.001
No	7505 (28.87)	2611 (27.90)	2629 (27.06)	63 (25.00)	1686 (33.59)	516 (31.29)	
Yes	18489 (71.13)	6748 (72.10)	7085 (72.94)	189 (75.00)	3334 (66.41)	1133 (68.71)	
FIR^#^							<0.001
Low (0–1.0)	4265 (16.41)	1559 (16.66)	1479 (15.23)	70 (27.78)	814 (16.22)	343 (20.80)	
Medium (1.1–3.0)	10736 (41.30)	3624 (38.72)	4044 (41.63)	107 (42.46)	2251 (44.84)	710 (43.06)	
High (>3.0)	10993 (42.29)	4176 (44.62)	4191 (43.14)	75 (29.76)	1955 (38.94)	596 (36.14)	
Marital status^#^							<0.001
Married	16822 (64.71)	6042 (64.56)	6451 (66.41)	163 (64.68)	3152 (62.79)	1014 (61.49)	
Separated	7113 (27.36)	2517 (26.89)	2588 (26.64)	68 (26.98)	1543 (30.74)	397 (24.08)	
Never married	2059 (7.92)	800 (8.55)	675 (6.95)	21 (8.33)	325 (6.47)	238 (14.43)	
Family history of diabetes or CVD^#^							<0.001
No	12512 (48.13)	4937 (52.75)	4761 (49.01)	111 (44.05)	2110 (42.03)	593 (35.96)	
Yes	13482 (51.87)	4422 (47.25)	4953 (50.99)	141 (55.95)	2910 (57.97)	1056 (64.04)	
History of diabetes^#^							<0.001
No	20900 (80.40)	8537 (91.22)	7848 (80.79)	187 (74.21)	3260 (64.94)	1068 (64.77)	
Yes	5094 (19.60)	822 (8.78)	1866 (19.21)	65 (25.79)	1760 (35.06)	581 (35.23)	
History of hypertension^#^							<0.001
No	12442 (47.86)	5717 (61.09)	4397 (45.26)	115 (45.63)	1588 (31.63)	625 (37.90)	
Yes	13552 (52.14)	3642 (38.91)	5317 (54.74)	137 (54.37)	3432 (68.37)	1024 (62.10)	
History of CHD^#^							<0.001
No	23363 (89.88)	8741 (93.40)	8674 (89.29)	229 (90.87)	4259 (84.84)	1460 (88.54)	
Yes	2631 (10.12)	618 (6.60)	1040 (10.71)	23 (9.13)	761 (15.16)	189 (11.46)	
History of stroke^#^							<0.001
No	24943 (95.96)	9065 (96.86)	9325 (96.00)	240 (95.24)	4737 (94.36)	1576 (95.57)	
Yes	1051 (4.04)	294 (3.14)	389 (4.00)	12 (4.76)	283 (5.64)	73 (4.43)	
Cardiometabolic diseases^#^							<0.001
No	24743 (95.19)	9161 (97.88)	9278 (95.51)	235 (93.25)	4547 (90.58)	4547 (90.58)	
Yes	1251 (4.81)	198 (2.12)	436 (4.49)	17 (6.75)	473 (9.42)	473 (9.42)	

MET, metabolic equivalent; HEI, healthy eating index; BMI, body mass index; FIR, Family income–poverty ratio; CVD, cardiovascular disease; CHD, coronary heart disease.

^※^These variables were expressed as mean ± SD and analyzed using analysis of variance.

^#^These variables were analyzed using the chi-square test.

^§^These variables were express as median (P_25_, P_75_) and analyzed using Kruskal-Wallis test.

### Associations of weight changes across adulthood with the incidence of diabetes

**Table 2** shows that compared to a stable normal pattern, a stable obesity pattern was related to a 358.0% (*HR*: 4.58, *95% CI*: 4.57, 4.58) higher risk of diabetes. Non-obesity to obesity pattern was associated with a 247.0% higher risk of incident diabetes (*HR*: 3.47, *95% CI*: 3.47, 3.47). Participants with maximum overweight had an 84.0% higher incidence of diabetes (*HR*: 1.84, *95% CI*: 1.84, 1.85).

**Table 2:** Associations of weight change across adulthood with the incidence of diabetes in NHANES 1999-2018.

Weight change	No. of incident diabetes/person-years	*HR*	*95% CI*	*P*
Weight change patterns^※^				
Stable normal	615/9169	*Ref*		
Maximum overweight	1313/9186	1.84	1.84,1.85	<0.001
Obesity to non-obesity	34/221	1.61	1.61,1.61	<0.001
Non-obesity to obesity	1131/4404	3.47	3.47,3.47	<0.001
Stable obesity	388/1459	4.58	4.57,4.58	<0.001
Absolute weight change ^#^				
Weight loss ≥ 2.5 kg	149/1389	1.19	1.19,1.20	<0.001
Weight change within 2.5 kg	562/6698	*Ref*		
Weight gain ≥ 2.5 and <10 kg	859/7564	1.22	1.22,1.22	<0.001
Weight gain ≥ 10 and < 20 kg	944/5308	1.89	1.89,1.90	<0.001
Weight gain ≥ 20 kg	967/3480	3.02	3.02,3.02	<0.001

^※^Adjustment for age, sex, race, education level, current smoking, current alcohol drinking, family income-poverty ratio level, marital status, family history of diabetes or CVD, MET, healthy eating index scores, and history of hypertension, CVD, and stroke.

^#^Adjustment for age, sex, height at examination, race, education level, current smoking, current alcohol drinking, family income-poverty ratio level, marital status, family history of diabetes or CVD, MET, healthy eating index scores, and history of hypertension, CVD, and stroke.

**Figure 3:**
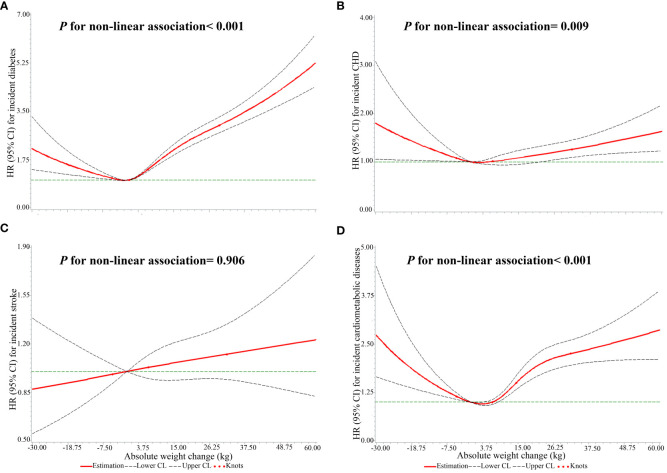
The non-linear associations of absolute weight changes across adulthood with the risks of single CMDs and CMM. **(A)** the outcome was diabetes; **(B)** the outcome was CHD; **(C)** the outcome was stroke; **(D)** the outcome was CMM.

When absolute weight change was a continuous variable, a J-shaped association for incident diabetes with absolute weight change was found (*P* for non-linear association< 0.001, **Figure 3A**). When classified into categories (**Table 2**), compared to weight change within 2.5 kg, weight loss and gain were linked to a higher incidence of diabetes. Furthermore, the extreme weight gain (weight gain≥ 20 kg) group had the largest effect on incident diabetes (*HR*: 3.02, *95% CI*: 3.02, 3.02).

### Associations of weight changes across adulthood with the incidence of CHD

Compared to stable normal patterns, maximum overweight, obesity to non-obesity, non-obesity to obesity, and stable obesity patterns were related to a higher incidence of CHD (**Table 3**). The stable obesity pattern had a stronger association with incident CHD than other weight change patterns (*HR*: 1.88, *95% CI*: 1.88, 1.88).

**Table 3:** Associations of weight change across adulthood with the incidence of CHD in NHANES 1999-2018.

Weight change	No. of incident CHD/person-years	*HR*	*95% CI*	*P*
Weight change patterns ^※^				
Stable normal	377/9123	*Ref*		
Maximum overweight	605/9292	1.31	1.31,1.31	<0.001
Obesity to non-obesity	17/246	1.58	1.58,1.59	<0.001
Non-obesity to obesity	468/4743	1.48	1.48,1.48	<0.001
Stable obesity	139/1606	1.88	1.88,1.88	<0.001
Absolute weight change^#^				
Weight loss ≥ 2.5 kg	101/1442	1.22	1.22,1.22	<0.001
Weight change within 2.5 kg	309/6711	*Ref*		
Weight gain ≥ 2.5 and <10 kg	404/7618	1.08	1.07,1.08	<0.001
Weight gain ≥ 10 and < 20 kg	406/5420	1.26	1.26,1.26	<0.001
Weight gain ≥ 20 kg	386/3819	1.37	1.37,1.37	<0.001

^※^Adjustment for age, sex, race, education level, current smoking, current alcohol drinking, family income-poverty ratio level, marital status, family history of diabetes or CVD, MET, healthy eating index scores, and history of hypertension, diabetes, and stroke.

^#^Adjustment for age, sex, height at examination, race, education level, current smoking, current alcohol drinking, family income-poverty ratio level, marital status, family history of diabetes or CVD, MET, healthy eating index scores, and history of hypertension, diabetes, and stroke.

There was a J-shaped association of absolute weight change with the incidence of CHD (*P* for non-linear association= 0.009, **Figure 3B**). Meanwhile, weight loss and gain were related to a higher risk of CHD (**Table 3**). Furthermore, the extreme weight gain (weight gain≥ 20 kg) group had the largest effect on incident CHD (*HR*: 1.37, *95% CI*: 1.37, 1.37).

### Associations of weight changes across adulthood with the incidence of stroke

**Table 4** shows that stable obesity participants had an increased risk of stroke (*HR*: 1.02, *95% CI*: 1.02, 1.03). Non-obesity to obesity and obesity to non-obesity patterns were respectively related to a 13.0% (*HR*: 1.13, *95% CI*: 1.13, 1.14) and 71.0% (*HR*: 1.71, *95% CI*: 1.70, 1.71) higher incidence of stroke. However, the maximum overweight group showed a reverse association with incident stroke (*HR*: 0.85, *95% CI*: 0.85, 0.86).

**Table 4:** Associations of weight change across adulthood with the incidence of stroke in NHANES 1999-2018.

Weight change	No. of incident stroke/person-years	*HR*	*95% CI*	*P*
Weight change patterns ^※^				
Stable normal	216/9307	*Ref*		
Maximum overweight	285/9688	0.85	0.85,0.86	<0.001
Obesity to non-obesity	8/250	1.71	1.70,1.71	<0.001
Non-obesity to obesity	233/5080	1.13	1.13,1.14	<0.001
Stable obesity	59/1669	1.02	1.02,1.03	<0.001
Absolute weight change^#^				
Weight loss ≥ 2.5 kg	40/1488	0.90	0.90,0.91	<0.001
Weight change within 2.5 kg	149/6863	*Ref*		
Weight gain ≥ 2.5 and <10 kg	217/7859	0.93	0.93,0.93	<0.001
Weight gain ≥ 10 and < 20 kg	205/5709	0.98	0.98,0.98	<0.001
Weight gain ≥ 20 kg	190/4075	1.12	1.11,1.12	<0.001

^※^Adjustment for age, sex, race, education level, current smoking, current alcohol drinking, family income-poverty ratio level, marital status, family history of diabetes or CVD, MET, healthy eating index scores, and history of hypertension, diabetes, and CVD.

^#^Adjustment for age, sex, height at examination, race, education level, current smoking, current alcohol drinking, family income-poverty ratio level, marital status, family history of diabetes or CVD, MET, healthy eating index scores, and history of hypertension, diabetes, and CVD.

As a continuous variable, there was no significant non-linear association of absolute weight change with the risk of stroke (*P* for non-linear association= 0.906, **Figure 3C**). With absolute weight change as a categorized variable, participants who lost weight more than 2.5 kg had a decreased risk of stroke, with a hazard ratio of 0.90 (95% CI: 0.90, 0.91). Small to moderate weight gain (weight gain ≥2.5 kg and <10 kg) and moderate to large weight gain (weight gain ≥10 kg and <20 kg) were also linked to a lower risk of stroke, with hazard ratios of 0.93 (95% CI: 0.93, 0.93) and 0.98 (95% CI: 0.98, 0.98). However, the extreme weight gain (weight gain ≥20 kg) group was related to an increased risk of stroke (HR: 1.12, 95% CI: 1.11, 1.12), as shown in **Table 4**.

### Associations of weight changes across adulthood with the risk of CMM

**Supplementary Table 2** presents that overweight and obesity at age 25 years were linked to a higher risk of CMM than underweight or normal weight (*HR*: 1.72 and 2.61, *95% CI*: 1.72, 1.72 and 2.61, 2.61). Similarly, participants with overweight or obesity at 10 years before recruitment had a higher risk of CMM (*HR*: 1.54 and 3.04, *95% CI*: 1.53, 1.54 and 3.04, 3.05).

**Table 5** displays the associations of weight change across adulthood with the risk of CMM. Stable obesity participants had 3.92 times the risk of CMM than stable normal ones (*HR*: 3.92, *95% C*I: 3.91, 3.92). Meanwhile, non-obesity to obesity was related to a 188.0% higher risk of CMM (*HR*: 2.88, *95% CI*: 2.88, 2.89), and obesity to non-obesity was related to a 179.0% higher risk of CMM (*HR*: 2.79, *95% CI*: 2.78, 2.80). The maximum overweight group was linked to a 50.0% higher risk of CMM (*HR*: 1.50, *95% CI*: 1.50, 1.50).

**Table 5:** Associations of weight change across adulthood with the risk of CMM in NHANES 1999-2018.

Weight change	No. of CMM/person-years	*HR*	*95% CI*	*P*
Weight change patterns^※^				
Stable normal	198/9359	*Ref*		
Maximum overweight	436/9714	1.50	1.50,1.50	<0.001
Obesity to non-obesity	17/252	2.79	2.78,2.80	<0.001
Non-obesity to obesity	473/5020	2.88	2.88,2.89	<0.001
Stable obesity	127/1649	3.92	3.91,3.92	<0.001
Absolute weight change^#^				
Weight loss ≥ 2.5 kg	71/1490	1.66	1.65,1.66	<0.001
Weight change within 2.5 kg	176/6896	*Ref*		
Weight gain ≥ 2.5 and <10 kg	268/7887	1.18	1.18,1.19	<0.001
Weight gain ≥ 10 and < 20 kg	335/5693	1.69	1.69,1.69	<0.001
Weight gain ≥ 20 kg	401/4028	2.78	2.78,2.78	<0.001

^※^ Adjustment for age, sex, race, education level, current smoking, current alcohol drinking, family income-poverty ratio level, marital status, family history of diabetes or CVD, MET, healthy eating index scores, and history of hypertension.

^#^Adjustment for age, sex, height at examination, race, education level, current smoking, current alcohol drinking, family income-poverty ratio level, marital status, family history of diabetes or CVD, MET, healthy eating index scores, and history of hypertension.

Meanwhile, a U-shaped relationship between absolute weight change and the risk of CMM was found (*P* for non-linear association< 0.001, **Figure 3D**). Participants with weight loss ≥ 2.5 kg had a 66.0% higher risk of CMM than weight change within 2.5 kg (*HR*: 1.66, *95% CI*: 1.65, 1.66), as shown in **Table 5**. Meanwhile, all weight gain groups (≥ 2.5 kg) were linked to an increased risk of CMM. Furthermore, the extreme weight gain (weight gain ≥20 kg) group had a stronger association with the risk of CMM (*HR*: 2.78, *95% CI*: 2.78, 2.78).

**Figure 4:**
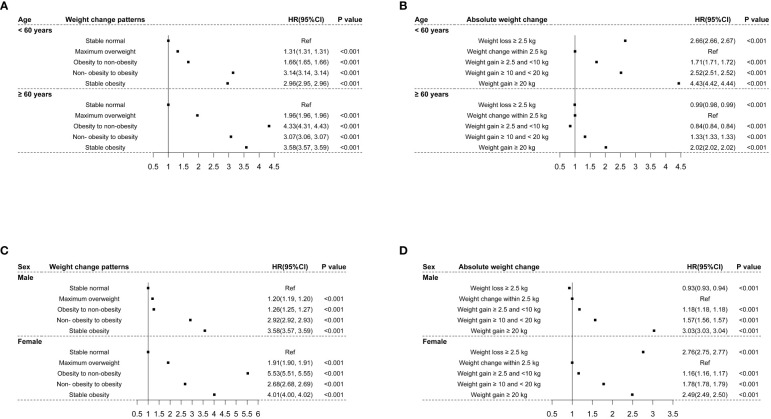
The associations of weight changes across adulthood with the risk of CMM stratified by age and sex. **(A)** indicated the associations of weight change patterns with the risk of CMM stratified by age; **(B)** indicated the associations of absolute weight change with the risk of CMM stratified by age; **(C)** indicated the associations of weight change patterns with the risk of CMM stratified by sex; **(D)** indicated the associations of absolute weight change with the risk of CMM stratified by sex.

In the stratified analyses, similar relationships between weight change patterns and the risk of CMM were observed in participants aged < 60 years and ≥ 60 years, and the effect size of weight change patterns on the risk of CMM was greater among participants aged≥ 60 years at baseline than their counterparts (**Figure 4A**). Meanwhile, the association of absolute weight change with the risk of CMM among participants < 60 years was consistent with the main results. However, among participants aged ≥ 60 years, weight loss≥ 2.5 kg and weight gain ≥2.5 kg and <10 kg were related to a lower risk of CMM (*HR*: 0.99 and 0.84, *95% CI*: 0.98, 0.94 and 0.84, 0.84), as shown in **Figure 4B**. On the other hand, similar relationships between weight change patterns and the risk of CMM were observed in men and women, and the effect sizes were greater among women than men (**Figure 4C**). The association of absolute weight change with the risk of CMM in females was comparable with the main results. However, weight loss≥ 2.5 kg was linked to a decreased risk of CMM (*HR*: 0.93, *95% CI*: 0.93, 0.94) in males, as shown in **Figure 4D**.

### Sensitivity analysis

When all variables were imputed using multiple imputations, the imputed dataset was used to repeat the relationships between weight change across adulthood and the risk of CMM. The results of sensitivity were comparable with the main results, as shown in **Supplementary Table 3**. As removing participants with new-onset CMM within two years after baseline, the associations of weight change with the risk of CMM were consistent with the main results, as shown in **Supplementary Table 4**.

## Discussion

A national retrospective cohort study was designed to evaluate the associations of weight changes across adulthood with the risks of single CMDs and CMM. Compared to a stable normal pattern, maximum overweight, obesity to non-obesity, non- obesity to obesity, and stable obesity were related to increased risks of diabetes, CHD, and CMM. However, the maximum overweight pattern was reversely associated with incident stroke. Compared to weight change within 2.5 kg, weight loss≥ 2.5 kg and weight gain≥ 2.5 kg were linked to higher risks of diabetes, CHD, and CMM. Whereas, weight loss≥ 2.5 kg and small to large weight gain (weight gain≥ 2.5 kg and < 20 kg) were related to a lower risk of stroke. Furthermore, J shaped or U shaped associations of absolute weight change with the risks of diabetes, CHD, and CMM, but not stroke, were observed.

This study examined the relationships between weight changes across adulthood and the incidence of single CMDs, and found that both weight gain and loss were positively associated with the risk of diabetes and CHD. Previous studies found that weight in early adulthood can influence subsequent diabetes risk (17, 18). Furthermore, weight change and weight gain during early adulthood have also been demonstrated to have a strong impact on the subsequent diabetes risk (19, 20). However, evidence on the relationship between weight loss and the risk of diabetes were mixed. Some studies suggested a reversed link between weight loss and incident diabetes, but others failed (21, 22). A recent study reported that weight loss≥ 2.5 kg was related to a 27% lower risk of diabetes, but any account of weight gain was positively associated with incident diabetes (1). On the other hand, weight gain can contribute to the development of CVD (23). Any account of weight gain can contribute to subsequent CVD risk, which is consistent with the findings of this study (1). It was documented that weight gain in early adulthood can lead to low-grade inflammation, a lower adiponectin level, and a higher leptin level, which can accelerate vascular aging and contribute to CVD (24).

In this study, the maximum overweight pattern was related to a 14.6% lower risk of stroke than a stable normal pattern, and reverse associations of weight loss and weight gain ≥ 2.5 and < 20 kg with the incident stroke were observed. However, a previous study reported that there was a positive link between weight gain and stroke in healthy men (25). Meanwhile, in a prospective study of participants aged 40-69 years, 5-year weight gain and weight loss were related to a higher risk of stroke only in women (26). The different results may be attributed to the different populations and cut-off values used to define weight change. The results of the restricted cubic spline showed the risk of stroke increased with increasing the magnitude of weight gain, which implied that there might be a threshold value effect of weight gain on the risk of stroke. Meanwhile, the time interval between age 25 years and 10 years before recruitment was relatively long (about 22 years). Weight change patterns only captured the weight at the two time points of age 25 years and 10 years before recruitment. It was possible that other weight change patterns occurred during this period of adulthood, for example losing and regaining weight (“weight cycling”). On the other hand, the obesity-stroke paradox might be one of the reasons. This paradox emphasizes the biological protective role of the adipose tissue, which is currently recognized as an endocrine organ (27). Soluble tumor necrosis factor-α (TNF-α) receptors secreted by adipose tissue may neutralize the biological impact of TNF-α, and contrast the post-stroke pro-inflammatory state (27). Moreover, a previous study on patients with dilated cardiomyopathy found that lower plasma adiponectin and increased leptin resistance were observed in obese patients (28). Therefore, the protective effect of weight gain on stroke should be viewed with caution and further examined in future study.

Up to now, little was known about the association of weight change across adulthood with the risk of CMM. A recent study reported that BMI severity over 20 years was linked to poor cardiometabolic health (29). A study using the data of NHANES reported that any weight change patterns were linked to accelerated biological aging compared with stable normal weight (15). It is documented that biological aging is closely related to health risks, including CMM. Meanwhile, another study using the data of NHANES found that maintaining a stable normal weight had the lowest risk of mortality, but stable obesity, weight gain, and weight loss were accompanied by an increased risk of mortality (13). Meanwhile, J-shaped or U-shaped associations of weight change with all-cause mortality were observed. These findings partly supported the results of this study that there were J-shaped associations of absolute weight change with the risks of diabetes and CHD and a U-shaped association of absolute weight change with the risk of CMM.

### Limitations and strengths

The strengths of the present study included the large and nationally representative sample of the US population and the solid evidence on the associations of weight changes from early to middle adulthood with the risks of single CMDs and CMM. However, the limitations of this study should also be clarified. First, there may be recall bias when participants were required to recall weights at age 25 years and 10 years before recruitment, though recalled weight has been validated to be close to measured weight (30). Second, the approximation of middle adulthood (age range: 26-75 years) was close to age 25 years. It may be indistinguishable between early and middle adulthood. Third, the mechanisms underlying the associations of weight changes with the risk of CMM were not fully explained. Fourth, given the data mining nature of second-hand data and retrospective epidemiological study, there could exist unmeasured or unavailable variables, such as other concomitant diseases including cancer, anorexia nervosa, bariatric surgery, and so on. These concomitant diseases were not considered or mentioned in this study but could confound the associations of weight change with the risks of CMDs and CMM. For example, weight loss was associated with an increased risk of diabetes in this study, which was inconsistent with previous studies (31–34). Weight loss might not be abiogenetic and might be caused by other concomitant diseases. Thus, there might be bias in the associations of weight change with the risks of CMDs and CMM. Therefore, further well-designed study is needed to confirm the results of this study in the future.

In conclusion, this study suggested that maintaining a stable normal weight can benefit from the lowest risk of diabetes, CHD, and CMM. Stable obesity, weight loss, and weight gain from early to middle adulthood were related to increased risks of diabetes, CHD, and CMM. However, weight loss and light weight gain were linked to a decreased risk of stroke. In addition, J-shaped or U-shaped associations of absolute weight change with the risk of diabetes, CHD, and CMM were found. Therefore, this study highlighted the importance of public health initiatives to maintain a stable and normal weight and to prevent overweight and obesity across adulthood. Meanwhile, greater public health efforts should be made to control weight across adulthood as a minimum goal for young adults to prevent the development of single CMDs and CMM.

## Data availability statement

The datasets presented in this study can be found in online repositories. The names of the repository/repositories and accession number(s) can be found below: https://www.cdc.gov/nchs/index.htm.

## Ethics statement

The studies involving humans were approved by the NHANES Institutional Review Board (protocols Numbers: NHANES Protocol #2011-17 and NHANES Protocol #2018-01). The studies were conducted in accordance with the local legislation and institutional requirements. The participants provided their written informed consent to participate in this study.

## Author contributions

FZ: Writing – original draft. QZ: Data curation, Formal analysis, Writing – review & editing. HW: Data curation, Formal analysis, Writing – review & editing. KW: Data curation, Formal analysis, Investigation, Writing – review & editing. SK: Project administration, Writing – review & editing. PM: Project administration, Writing – review & editing. XW: Conceptualization, Writing – review & editing.
