# Supplementary material for: Weight changes from early to middle adulthood and cardiometabolic multimorbidity later in life among middle-aged and older adults: a retrospective cohort study from the NHANES 1999-2018
Source: Front Endocrinol (Lausanne). 2024 Feb 19;15:1306551. doi: 10.3389/fendo.2024.1306551 (PMC10910024; doi:10.3389/fendo.2024.1306551)
Supplement: Supplementary file 1 [file DataSheet_1.docx]

Supplementary Table 1 Characteristics of participants in the NHANES 1999–2018 according to absolute weight change from age 25 years to 10 years before recruitment

| Characteristics | Weight loss ≥ 2.5 kg  (n= 1490) | Weight change within 2.5 kg  (n= 6896) | Weight gain ≥ 2.5 and <10 kg  (n= 7887) | Weight gain ≥ 10 and < 20 kg  (n= 5693) | Weight gain ≥ 20 kg (n= 4028) | *P* |
| --- | --- | --- | --- | --- | --- | --- |
| Age (years) ^※^ | 47.13±14.48 | 42.04±14.03 | 46.95±13.01 | 50.68±12.38 | 52.01±11.64 | <0.001 |
| MET (MET-min/week)^§^ | 643 (14, 2700) | 720 (35, 2599) | 600(28, 2200) | 540 (7, 2039) | 339 (0, 1755) | <0.001 |
| HEI score^※^ | 52.80±14.55 | 52.30±13.94 | 53.88±13.98 | 53.71±13.35 | 53.08±13.26 | <0.001 |
| BMI at age 25 years  (kg/m^2^) ^※^ | 27.07±5.65 | 23.82±4.48 | 23.19±3.77 | 22.97±4.07 | 23.22±4.76 | <0.001 |
| BMI at 10 years before recruitment (kg/m^2^) ^※^ | 24.55±4.92 | 24.31±4.50 | 25.96±3.78 | 28.77±4.15 | 34.57±6.11 | <0.001 |
| Absolute weight change  (kg) ^※^ | -8.76±5.75 | 0.41±1.25 | 6.49±2.04 | 14.47±2.62 | 29.64±8.97 | <0.001 |
| Sex^＃^ |  |  |  |  |  | <0.001 |
| Male | 800(53.69) | 3645(52.86) | 4020(50.97) | 2752(48.34) | 1856(46.08) |  |
| Female | 690(46.31) | 3251(47.14) | 3867(49.03) | 2941(51.66) | 2172(53.92) |  |
| Race^＃^ |  |  |  |  |  | <0.001 |
| Mexican-American | 222(14.90) | 997(14.46) | 1210(15.34) | 880(15.46) | 553(13.73) |  |
| Other Hispanic | 107(7.18) | 467(6.77) | 602(7.63) | 432(7.59) | 277(6.88) |  |
| Non-Hispanic White | 732(49.13) | 3509(50.88) | 3964(50.26) | 2863(50.29) | 2029(50.37) |  |
| Non-Hispanic Black | 307(20.60) | 1293(18.75) | 1507(19.11) | 1174(20.62) | 995(24.70) |  |
| Other Race | 122(8.19) | 630(9.14) | 604(7.66) | 344(6.04) | 174(4.32) |  |
| Education level^＃^ |  |  |  |  |  | <0.001 |
| Less than high school | 433(29.06) | 1676(24.30) | 1821(23.09) | 1397(24.54) | 1055(26.19) |  |
| High school or equivalent | 376(25.23) | 1568(22.74) | 1844(23.38) | 1384(24.31) | 1010(25.07) |  |
| College or above | 681(45.70) | 3652(52.96) | 4222(53.53) | 2912(51.15) | 1963(48.73) |  |
| Current smoking^＃^ |  |  |  |  |  | <0.001 |
| No | 635(42.62) | 3362(48.75) | 4101(52.00) | 2884(50.66) | 1977(49.08) |  |
| Yes | 855(57.38) | 3534(51.25) | 3786(48.00) | 2809(49.34) | 2051(50.92) |  |
| Current alcohol drinking^＃^ |  |  |  |  |  | <0.001 |
| No | 423(28.39) | 1741(25.25) | 2142(27.16) | 1787(31.39) | 1412(35.05) |  |
| Yes | 1067(71.61) | 5155(74.75) | 5745(72.84) | 3906(68.61) | 2616(64.95) |  |
| FIR^＃^ |  |  |  |  |  | <0.001 |
| Low (0–1.0) | 341(22.89) | 1258(18.24) | 1194(15.14) | 825(14.49) | 647(16.06) |  |
| Medium (1.1–3.0) | 641(43.02) | 2783(40.36) | 3110(39.43) | 2380(41.81) | 1822(45.23) |  |
| High (>3.0) | 508(34.09) | 2855(41.40) | 3583(45.43) | 2488(43.70) | 1559(38.70) |  |
| Marital status^＃^ |  |  |  |  |  | <0.001 |
| Married | 872(58.52) | 4484(65.02) | 5252(66.59) | 3697(64.94) | 2517(62.49) |  |
| Separated | 450(30.20) | 1723(24.99) | 2056(26.07) | 1635(28.72) | 1249(31.01) |  |
| Never married | 168(11.28) | 689(9.99) | 579(7.34) | 361(6.34) | 262(6.50) |  |
| Family history of diabetes or CVD^＃^ |  |  |  |  |  | <0.001 |
| No | 761(51.07) | 3608(52.32) | 3870(49.07) | 2577(45.27) | 1696(42.11) |  |
| Yes | 729(48.93) | 3288(47.68) | 4017(50.93) | 3116(54.73) | 2332(57.89) |  |
| History of diabetes^＃^ |  |  |  |  |  | <0.001 |
| No | 1235(82.89) | 6123(88.79) | 6689(84.81) | 4351(76.43) | 2502(62.12) |  |
| Yes | 255(17.11) | 773(11.21) | 1198(15.19) | 1342(23.57) | 1526(37.88) |  |
| History of hypertension^＃^ |  |  |  |  |  | <0.001 |
| No | 786(52.75) | 4253(61.67) | 3983(50.50) | 2225(39.08) | 1195(29.67) |  |
| Yes | 704(47.25) | 2643(38.33) | 3904(49.50) | 3468(60.92) | 2833(70.33) |  |
| History of CHD^＃^ |  |  |  |  |  | <0.001 |
| No | 1337(89.73) | 6399(92.79) | 7205(91.35) | 5010(88.00) | 3412(84.71) |  |
| Yes | 153(10.27) | 497(7.21) | 682(8.65) | 683(12.00) | 616(15.29) |  |
| History of stroke^＃^ |  |  |  |  |  | <0.001 |
| No | 1430(95.97) | 6689(97.00) | 7602(96.39) | 5433(95.43) | 3789(94.07) |  |
| Yes | 60(4.03) | 207(3.00) | 285(3.61) | 260(4.57) | 239(5.93) |  |
| Cardiometabolic diseases^＃^ |  |  |  |  |  | <0.001 |
| No | 1419(95.23) | 6720(97.45) | 7619(96.60) | 5358(94.12) | 3627(90.04) |  |
| Yes | 71(4.77) | 176(2.55) | 268(3.40) | 335(5.88) | 401(9.96) |  |

MET: metabolic equivalent; HEI: healthy eating index; BMI: body mass index; FIR: Family income–poverty ratio; CVD: cardiovascular disease; CHD: coronary heart disease

^※^These variables were expressed as mean ± SD and analyzed using *analysis of variance*.

^＃^These variables were analyzed using the *chi-square* test*.*

^§^These variables were express as median (P_25_, P_75_) and analyzed using *Kruskal-Wallis* test.

Supplementary Table 2 Associations of body weight in the two periods with the risk of CMM^※^

| Body weight | *HR* | *95% CI* | *P* |
| --- | --- | --- | --- |
| **At the age of 25 years** |  |  |  |
| Underweight or normal weight | *Ref* |  |  |
| Overweight | 1.72 | 1.72,1.72 | <0.001 |
| Obesity | 2.61 | 2.61,2.61 | <0.001 |
| **At 10 years before recruitment** |  |  |  |
| Underweight or normal weight | *Ref* |  |  |
| Overweight | 1.54 | 1.53,1.54 | <0.001 |
| Obesity | 3.04 | 3.04,3.05 | <0.001 |

^※^Adjustment for age, sex, race, education level, current smoking, current alcohol drinking, family income-poverty ratio level, marital status, family history of diabetes or CVD, MET, healthy eating index scores, and history of hypertension.

Supplementary Table 3 Associations of weight change across adulthood with the risk of CMM in sensitivity analysis using multiple imputation dataset (N= 35301)

| Weight change | *HR* | *95% CI* | *P* |
| --- | --- | --- | --- |
| **Weight change patterns** ^※^ |  |  |  |
| Stable normal | *Ref* |  |  |
| Maximum overweight | 1.57 | 1.51,1.64 | <0.001 |
| Obesity to non-obesity | 3.36 | 3.00,3.71 | <0.001 |
| Non-obesity to obesity | 2.92 | 2.75,3.08 | <0.001 |
| Stable obesity | 3.83 | 3.55,4.12 | <0.001 |
| **Absolute weight change**^＃^ |  |  |  |
| Weight loss ≥ 2.5 kg | 1.69 | 1.68,1.71 | <0.001 |
| Weight change within 2.5 kg | *Ref* |  |  |
| Weight gain ≥ 2.5 and <10 kg | 1.11 | 1.10,1.13 | <0.001 |
| Weight gain ≥ 10 and < 20 kg | 1.68 | 1.66,1.70 | <0.001 |
| Weight gain ≥ 20 kg | 2.68 | 2.61,2.75 | <0.001 |

^※^ Adjustment for age, sex, race, education level, current smoking, current alcohol drinking, family income-poverty ratio level, marital status, family history of diabetes or CVD, MET, healthy eating index scores, and history of hypertension.

^＃^Adjustment for age, sex, height at examination, race, education level, current smoking, current alcohol drinking, family income-poverty ratio level, marital status, family history of diabetes or CVD, MET, healthy eating index scores, and history of hypertension.

Supplementary Table 4 Associations of weight change across adulthood with the risk of CMM in NHANES 1999-2018 with removing onset CMM participants within two years after baseline

| Weight change | *HR* | *95% CI* | *P* |
| --- | --- | --- | --- |
| **Weight change patterns**^※^ |  |  |  |
| Stable normal | *Ref* |  |  |
| Maximum overweight | 1.38 | 1.37,1.38 | <0.001 |
| Obesity to non-obesity | 2.82 | 2.81,2.83 | <0.001 |
| Non-obesity to obesity | 2.71 | 2.71,2.72 | <0.001 |
| Stable obesity | 3.47 | 3.46,3.47 | <0.001 |
| **Absolute weight change**^＃^ |  |  |  |
| Weight loss ≥ 2.5 kg | 1.71 | 1.71,1.71 | <0.001 |
| Weight change within 2.5 kg | *Ref* |  |  |
| Weight gain ≥ 2.5 and <10 kg | 1.23 | 1.23,1.23 | <0.001 |
| Weight gain ≥ 10 and < 20 kg | 1.70 | 1.70,1.70 | <0.001 |
| Weight gain ≥ 20 kg | 2.78 | 2.78,2.79 | <0.001 |

^※^ Adjustment for age, sex, race, education level, current smoking, current alcohol drinking, family income-poverty ratio level, marital status, family history of diabetes or CVD, MET, healthy eating index scores, and history of hypertension.

^＃^Adjustment for age, sex, height at examination, race, education level, current smoking, current alcohol drinking, family income-poverty ratio level, marital status, family history of diabetes or CVD, MET, healthy eating index scores, and history of hypertension.
